# Enhanced preservation of vacuum-packaged Atlantic salmon by hyperbaric storage at room temperature versus refrigeration

**DOI:** 10.1038/s41598-021-81047-4

**Published:** 2021-01-18

**Authors:** Liliana G. Fidalgo, Mário M. Q. Simões, Susana Casal, José A. Lopes-da-Silva, Ivonne Delgadillo, Jorge A. Saraiva

**Affiliations:** 1 LAQV-REQUIMTE, Department of Chemistry, University of Aveiro, 3810-193 Aveiro, Portugal; 2 LAQV-REQUIMTE, Department of Chemistry, Faculty of Pharmacy, University of Porto, Porto, Portugal

**Keywords:** Lipids, Chemical safety

## Abstract

Hyperbaric storage at room temperature (HS/RT: 75 MPa/25 °C) of vacuum-packaged fresh Atlantic salmon (*Salmo salar*) loins was studied for 30 days and compared to atmospheric pressure at refrigerated temperatures (AP/5 °C, 30 days) and RT (AP/25 °C, 5 days). Most of the fatty acids were not affected by storage conditions, with only a slight decrease of docosahexaenoic acid (DHA) content (n-3 polyunsaturated fatty acid) for AP samples, reflected in the lower polyene index values obtained and higher oxidation extent. For HS, a lower lipid oxidation extension and a slower increase of myofibrillar fragmentation index values were observed, when compared to AP samples. The volatile profile was similar for the HS and fresh samples, with the HS samples retaining fresh-like alcohols and aldehydes components, which disappeared in AP samples, mainly in AP/25 °C samples. The volatile profile for AP samples (5 and 25 °C) revealed mostly spoilage-like compounds due to microbial activity. Drip loss increased progressively during the 30 days of storage under HS, while a slight decrease of water holding capacity after 5 days was observed, increasing further after 30 days. Regarding textural properties, only resilience was affected by HS, decreasing after 30 days. So, HS/RT could represent an interesting extended preservation methodology of fresh salmon loins, since allows retaining important physicochemical properties for at least 15 days, while refrigeration after 5 days showed already volatile spoilage-like compounds due to microbial activity. Furthermore, this methodology allows additional considerable energy savings when compared to refrigeration.

## Introduction

Chilled fresh fish has a reduced shelf-life due to its high perishability. During chilled storage, deterioration of fresh fish results from the joint detrimental effects of microorganisms proliferation, enzymes action and lipid oxidation^1^. Oxidation decreases fish quality during storage by formation of unpleasant smells and lipid peroxides, resulting in flavour, texture and consistency losses, and leading to reduction of nutritional value^2^. Lipid oxidation vulnerability increases as the number of double bonds in the fatty acid increments, and thus polyunsaturated fatty acids (PUFAs) are very much prone to oxidation^3^. Atlantic salmon (*Salmo salar*) is a good source of PUFAs, particularly the important omega-3 fatty acids: eicosapentaenoic acid (EPA) and docosahexaenoic acid (DHA)^4^. Compared to other fishes, salmon has a higher oxidative stability^5^ due to the presence of astaxanthin, responsible for the characteristic red/orange coloration. The typical colour of salmon is also distinguishable characteristic for salmonids playing a pronounced effect on consumer product acceptability and price.

Hyperbaric storage (HS) is being increasingly studied in the last years since the results reported the likelihood to preserve foods under moderate pressure (up to ~ 100 MPa), with longer shelf-life and equal/better quality compared to the present-day commercial refrigeration. HS at room temperature (HS/RT) was already applied on fish products, proving its efficiency in extending shelf-life^6,7^. In a previous work, the effects of HS at 75 MPa/25 °C were reported for microbial proliferation and some physicochemical properties (such as colour and lipid oxidation) of Atlantic salmon^7^, being observed an increment of the microbial shelf-life up to a minimum of 25 days comparatively to refrigeration (3 days). In this work, salmon was stored in the presence of air, which reflected on the larger peroxide values and thiobarbituric acid-reactive substances (TBARS) for HS, compared to chilling, while an even higher and advanced lipid oxidation state was verified for samples preserved at atmospheric pressure and RT^7^.

Recently, Cape hake (*Merluccius* spp.) loins^8^ and Atlantic mackerel (*Scomber scombrus*, L.) fillets^9^ preservation was studied under HS at low temperature (50 MPa/5 °C). The results indicate microbial growth inhibition (no changes were observed comparatively to initial loads), after 7 and 12 days, respectively, with no significant lipid degradation observed on Atlantic mackerel fillets during the 12 days^9^. However, these studies were done under pressure but at low temperature of 5 °C, which still requires additional energy to control temperature during all storage time.

Thus, this work aimed studying the stability of vacuum-packaged Atlantic salmon preserved by HS at RT (75 MPa/25 °C), according to the best conditions obtained in a previous work^7^ regarding microbial stability, to ascertain if vacuum could decrease lipid oxidation observed in the previous work. Atlantic salmon samples were vacuum-packaged and evaluated regarding important physicochemical parameters during 30 days of storage, such as fatty acids profile, lipid oxidation, myofibrillar fragmentation index, physical parameters (drip loss, water holding capacity and texture) and volatile compounds.

It should be highlighted that previous studies^7^ of other quality parameters of salmon preserved by HS in the same conditions as used in the present work (75 MPa/25 °C)), like colour revealed no colour changes up to 25 days. The same previous work^7^ studied also salmon microbial preservation by HS at 75 MPa/25 °C (total aerobic and anaerobic mesophiles, *Enterobacteriaceae*, lactic acid bacteria, and *Pseudomonas* spp.), reporting a reduction of about 3.5 log units of initial microbial counts, leading to an increase of the microbial shelf-life of at least 25 days, compared to RF (3 days). This way the parameters studied in the cited previous work^7^ were not studied in the present work.

## Materials and methods

### Sample preparation

Atlantic salmon (*Salmo salar*) loins were purchased in a local market just before each experiment. The skin was removed under aseptic conditions, and loins with *ca.* 60 g were selected (size: 10 cm × 4 cm × 1.5 cm). Loins were selected from two individuals with similar weight (*ca.* 9 kg) and length (*ca.* 120 cm) and vacuum-packaged in low-oxygen permeable barrier bags (PA/PE-90; Plásticos Macar–Indústria de Plásticos Lda., Palmeira, Portugal) and sealed under vacuum at − 1 bar of pressure (Vacuum Packaging Machine Culinary, Albipack, Águeda, Portugal). To avoid deterioration, all samples were held on ice, and storage experiments were initiated immediately after sample preparation (within 2 h at most). Storage experiments took place between March and June 2018.

### Storage conditions

Pressure/temperature storage at 75 MPa/25 °C (this storage condition was selected according to the results obtained in a previous work^7^) was carried out up to 30 days. Vacuum-packaged salmon samples were put inside pressure vessels and pressure generated until reaching 75 MPa. After 5, 15 and 30 days, pressure was release and samples were taken for analysis. Control samples were preserved at atmospheric pressure (0.1 MPa, AP), at the same temperature (AP/25 °C, during 5 days) and under conventional refrigeration (AP/5 °C, during 30 days, except for fatty acids determination and lipid oxidation analyses, which was carried out only after 15 days due to samples scarcity at 30 days), in the same conditions (in the dark and submersed in the same fluid used for compression). Storage at AP/25 °C was only carried out for 5 days, because samples showed clear signs of high deterioration state.

HS was performed using a 2-L high pressure equipment (FPG7100, Stansted Fluid Power, Stansted, United Kingdom) equipped with a pressure vessel of 100-mm diameter and 250-mm height. This high-pressure equipment uses a mixture of propylene glycol and water (40:60, v/v) as pressurization fluid.

### Lipid hydrolysis and oxidation

#### Lipid extraction

The lipid fraction was extracted and quantified as described before^7^, following the method of Bligh and Dyer^10^ and used for the determination of fatty acids and peroxides values (primary lipid oxidation), being the upper layers used for fluorescence compounds quantification. Both quantifications were done just after lipid extraction (in the same day) with random sampling. The total extracted lipid content ranged from 5.49 to 10.22% (w/w), values in the ranged reported in the literature for the same fish species^11^.

#### Fatty acids determination

Determination of fatty acids was performed by gas chromatography. Synthesis of fatty acid methyl esters (FAMEs) was carried out accordingly to O’Fallon et al.^12^. To a tube containing 40 µg of oil that resulted from lipid extraction, 1 mL of a surrogate fatty acid (C17:0–4 mg/mL in methanol; with a surrogate recovery of 98% after FAMEs synthesis) was added, followed by 5.3 mL of methanol and 0.7 mL of 10 N potassium hydroxide. After vortex-mixing for 10 s, the tube was incubated in a thermostatic bath at 55 °C with stirring for 1.5 h. The mixture was homogenized by inversion and 0.58 mL of 12 mol/L sulphuric acid were added, occurring the formation of a white precipitate (potassium sulphate). The tube was incubated again at 55 °C for 1.5 h and then cooled again in cold water, thus occurring the FAMEs synthesis. Then, 3 mL of hexane were added and vortex-mixed for 5 min. After centrifugation for 5 min (3000 rpm), the hexane layer, containing the FAMEs, was transferred to another tube, which already contained 0.5 g of anhydrous sodium sulphate. After another centrifugation for 5 min, the hexane layer (900 µL), containing the FAMEs, was placed into a GC vial and 100 µL of the internal standard C19:0 (10 mg/mL of C19:0 methyl ester in hexane) were added.

FAMEs were analysed using a gas chromatograph mass spectrometer (GC–MS Shimadzu QP2010 Ultra) equipped with an AOC-20i autosampler (Shimadzu, Japan), with the electron impact ionization (EI) at 70 eV and high-performance quadrupole mass filter. The separation of the compounds was carried out on a fused-silica DB-5 MS type capillary column (30 m length, 0.25 mm i.d., 0.25 µm film thickness) using helium as the carrier gas (40 cm/s) and a split ratio = 50. The GC–MS chromatographic conditions were as follows: injection port temperature 300 °C; initial oven temperature 140 °C (0 min), rising to 250 °C (0 min) at 1.0 °C/min and then rising to 280 °C (for 2 min) at 10.0 °C/min. The mass spectrometer was operated over a range of m/z 50–1000. The ion source was kept at 200 °C and the interface temperature at 300 °C. Peaks were automatically integrated (GCMS solution Version 4.20), C19:0 fatty acid methyl ester being used as internal standard for quantitative analysis. Peaks were identified by comparison of their retention times with standard FAME mixtures (Supelco 37 FAME Mix, Sigma-Aldrich, Missouri, EUA), and the compound/internal standard signal ratio for each peak was used for the calculation of concentrations, using different calibration curves of standard FAME mixtures. Three salmon samples were used for each storage condition with a duplicate GC injection. Results are shown as g of fatty acids (g FA)/100 g of lipids.

Also, the polyene index (PI), as a ratio of polyunsaturated to saturated fatty acids, was calculated as follows^13^:

$$\text{Polyene}\; \text{index}=\frac{\text{ C}20{:}5 +\text{ C}22{:}6}{\text{C}16{:}0}$$

PI is usually used to evaluate lipid oxidation in fish products, being a measure of PUFAs damage, since it quantifies the variation of LC‐PUFAs (C20:5 and C22:6), relative to a saturated fatty acid representative of marine products such as salmon (C16:0).

#### Lipid oxidation

Peroxide value determination (primary lipid oxidation) was carried out following the method used by Gheisari et al.^14^, and already described in a previous work^7^. The peroxide value was expressed as mg Fe III/kg lipids. Secondary lipid oxidation was evaluated by quantification of secondary lipid oxidation products using the thiobarbituric acid-reactive substances (TBARS) method, which was performed accordingly to Vyncke^15^ and as previously described^7^. TBARS results were expressed as µg malondialdehyde (MDA)/g fish. Tertiary lipid oxidation compounds resulting from the interaction between oxidized lipids and nucleophilic compounds (namely, protein-like molecules) were measured by fluorescence spectroscopy (Hitachi F2000 fluorescence spectrophotometer (Tokyo, Japan). In agreement with previous research^16^, fluorescence measurements were carried out at 393/463 nm and 327/415 nm in the aqueous phase (methanol–water layer) resulting from the lipid extraction of fish muscle^10^. The relative fluorescence (RF)^15^ was calculated as follows: $RF=F/{F}_{st}$, where F is the fluorescence measured at each excitation/emission wavelength pair and ${F}_{st}$ is the fluorescence intensity of a quinine sulphate solution (1 µg/mL in 0.05 M H_2_SO_4_) at the corresponding wavelength pair. The fluorescence ratio (FR) was calculated as the ratio between the two RF values:

$$FR= \frac{{RF}_{\frac{393 \;\text{nm}}{463 \;\text{nm}}}}{{RF}_{\frac{237 \;\text{nm}}{415 \;\text{nm}}}}$$

### Protein stability–myofibrillar fragmentation index

Myofibrillar fragmentation index (MFI) was determined by the method of Hopkins with slight modifications^17^. Muscle tissue was pulverized in liquid nitrogen and 0.5 g of this powdered tissue was homogenized for 1 min (MICCRA D-9 Homogenizer, MICCRA GmbH, Müllheim, Germany) in 30 mL of 25 mM phosphate buffer (0.1 M potassium chloride, 1 mM EDTA, pH 7.0). The suspension was filtered to remove connective tissue and the residue was washed with 10 mL of the same phosphate buffer. Then, filtrate was centrifuged at 1000×*g* for 15 min at 4 °C (Heraeus Biofuge Stratos, Thermo, Electron Corporation, Massachusetts, EUA), the precipitate was resuspended in 10 mL of phosphate buffer and centrifuged again. This step was repeated twice and the pellet was resuspended in buffer solution (10 mL). Protein concentrations were determined and after adjustment to a concentration of 0.5 mg/mL, using the same buffer, absorbance measurements at 540 nm were done (Multiskan Go microplate spectrophotometer, Thermo Scientific, Waltham, EUA). Protein concentrations were determined by the Bradford assay modified by Zor and Selinger^18^ and using bovine serum albumin (BSA) as standard (0.1–0.6 mg BSA/mL of phosphate buffer). MFI was calculated by multiplying measurements with 150.

### Volatile compounds characterization by HS-SPME-GC/MS

The characterization of the volatile fraction of the salmon samples was performed by headspace solid-phase microextraction (HS-SPME) gas-chromatography-mass spectrometry (GC/MS). Salmon samples (2 g) were cut and weighed into a vial containing a micro stirring bar. Then, 1 mL of internal standard (0.473 mg/mL cyclohexanone in water), 3 mL of ultrapure water and 1.44 g of sodium chloride were added into the vial and sealed with a silicone septum. The sample was equilibrated for 20 min at 50 °C followed by HS-SPME exposure at the same temperature under stirring (500×*g*). The volatile compounds in the headspace were absorbed onto SPME fibres for 40 min based on (DVB/CAR/PDMS 50/30 mm; Supelco, Bellefonte, USA) for 40 min. Salmon samples were analysed in triplicate for each condition. The retained compounds were thermally desorbed from the fibre for 5 min in the injection port (splitless mode; 250 °C). The fibre was maintained for further 5 min in the injector port of the chromatography system for cleaning and conditioning for further analyses.

Chromatographic separation was performed on a fused-silica DB-5 MS Capillary GC column (30 m × 0.25 mm I.D. × 0.25 μm film thickness, Agilent) with a temperature program from 40 to 235 °C, with a total run time of 60 min. The MS transfer line and ion source were at 280 °C and 230 °C respectively, and MS quadrupole temperature at 150 °C, with electron ionization of 70 eV; set in full scan mode (m/z 40 to 650 at 1.2 scan/s). Identification of the volatile compounds detected by GC/MS analysis was based on computer matching with the reference mass spectra of the MS library of NIST 11 and Wiley 7.0, retention times (RTs) and retention index (RIs). Semi-quantitative determinations were carried out by using cyclohexanone as an internal standard. Volatile compounds content was calculated by comparing the peak area of each compound with that of the internal standard.

### Physical properties

#### Drip loss and water holding capacity

Drip loss was quantified as the relative weight between day 0 (${m}_{0}$) and day × (${m}_{x}$):

$$Drip loss (\%)=\frac{{m}_{0}-{m}_{x}}{{m}_{0}}\times 100$$

Water holding capacity (WHC) was measured by the method of Szajdek and Borowska^19^. Briefly, a piece of salmon previously weighted (*ca* 1 g) was wrapped in two filter papers (also weighted; Whatman #1) and centrifuged (530×*g*, 15 min, 4 °C; Heraeus Biofuge Stratos, Thermo, Electron Corporation, Massachusetts, EUA) and, after centrifugation, the sample was removed, and the filter papers weighted. WHC was calculated using the following equations:

$$WHC=\frac{{W}_{0}-\Delta W}{{W}_{0}}\times 100$$

which $${W}_{0}=\frac{{V}_{0}}{\left({V}_{0}+{D}_{0}\right)}\times 100$$ and $$\Delta W=\frac{{\Delta V}_{0}}{({V}_{0}+{D}_{0})}\times 100$$.

The initial weight of raw sample ${m}_{0}$ was equal to the sum of the initial water content of raw material, ${V}_{0}$, and dry material, ${D}_{0}$, being estimated by drying previously weighed salmon samples (*ca.* 5 g) at 103 ± 2 °C during 24 h^20^. The weight of the exudates (the liquid separated from the sample during centrifugation) is named ${\Delta V}_{0}$. The solid contents of the exudates are regarded as negligible in these calculations. All determinations were carried out in triplicate of salmon samples for each condition.

#### Texture profile analyses (TPA)

Texture profile analyses (TPA) of salmon samples were carried out perpendicular to the myotomes. Measurements were taken with a cylindrical probe 6-mm diameter fitted to a TA.HDi texture analyser (Stable Micro Systems, Surrey, England) equipped with a 5 kg load cell. The crosshead moved at a constant speed of 2 mm/s. The test conditions were two consecutive cycles with 5 s between cycles and 1 cm of penetration.

From the resulting force–time curve, the following parameters were determined: *hardness* (N), maximum force required to compress the sample; *adhesiveness* (N.s), the largest negative force value during the upstroke; *springiness* or *elasticity*, sample ability to recover its original form after the deforming force is removed; and *resilience*, area of the first upstroke relative to the area of the first downstroke.

### Statistical analysis

Data were tested with one-way analysis of variance (ANOVA), followed by a multiple comparisons test (Tukey’s Honestly Significant Difference, HSD) to identify differences between conditions and during storage period. The level of significance was established at p < 0.05.

## Results and discussion

### Lipid stability under HS of salmon loins: fatty acids profile and lipid oxidation

#### Fatty acids profile evolution

Fifteen fatty acids (FA) were identified in the salmon samples under study, as can be seen in Table 1. The FA composition did not change significantly between samples, showing a moderate content of polyunsaturated fatty acids (PUFAs, 17.6–22.1 g/100 g lipids), with a low amount of saturated fatty acids (SFA, 9.6–11.6 g/100 g lipids) and a high content of monounsaturated fatty acids (MUFA, 37.4–42.4 g/100 g lipids). The PUFA/SFA ratio was between 1.77 and 1.97, which were in all the cases higher than the minimum recommended value for human diet of 0.45^21^.

**Table 1:** Fatty acids (FA) profile (wt.%, g FA/100 g of total lipids) of fresh Atlantic salmon (for values along storage see Table 1S).

Free fatty acids	Fresh fish
	0 days
C14:0	1.14 ± 0.12
C16:0	7.57 ± 1.04
C18:0	2.05 ± 0.16
**∑ SFA**	**10.76 ± 1.22**
C16:1n-7	1.59 ± 0.28
C18:1n-7	3.56 ± 0.55
C18:1n-9	29.29 ± 7.11
C20:1n-9	4.84 ± 0.58
C22:1n-9	0.64 ± 0.06
**∑ MUFA**	**39.91 ± 8.45**
C20:4n-3	0.74 ± 0.10
C20:5n-3 (EPA)	1.98 ± 0.16
C22:5n-3	0.94 ± 0.11
C22:6n-3 (DHA)	4.51 ± 0.70
**∑ n-3 PUFA**	**8.17 ± 0.89**
C18:2n-6	9.04 ± 1.63
C20:2n-6	0.95 ± 0.15
C22:2n-6	2.24 ± 0.45
**∑ n-6 PUFA**	**12.23 ± 2.12**
**PUFA/SFA**	**1.90 ± 0.17**
**n-6/n-3 ratio**	**1.51 ± 0.27**

SFA: total saturated fatty acid; MUFA: monounsaturated fatty acid; PUFA: polyunsaturated fatty acid; EPA: Eicosapentaenoic acid; DHA: Docosahexaenoic acid.

The total SFA composition was not significantly (p > 0.05) affected by storage (Table 1S). The source of total SFAs mainly came from myristic acid (C14:0), palmitic acid (C16:0) and stearic acid (C18:0), with FA contents in the initial salmon samples of 1.14 ± 0.12, 7.57 ± 1.04 and 2.05 ± 0.16 g/100 g lipids, respectively. Among SFAs, palmitic acid (C16:0) was largely predominant in all samples (7.15–8.71 g/100 g lipids), followed by stearic acid (C18:0) (1.57–2.06 g/100 g lipids). Similar results were observed for other fish species, such as saithe (*Pollachius virens*) and hoki (*Macruronus novaezelandiae*)^22^, and Atlantic mackerel (*Scomber scombrus*)^23^.

MUFA predominated in the FA profile (Table 1), with palmitoleic acid (C16:1n-7), vaccenic acid (C18:1n-7), oleic acid (C18:1n-9), gadoleic acid (C20:1n-9) and eruric acid (C22:1n-9) showing initial values of 1.59 ± 0.28, 3.56 ± 0.55, 29.29 ± 7.11, 4.84 ± 0.58 and 0.64 ± 0.06 g/100 g lipids, respectively. Among the MUFAs, oleic acid (C18:1n-9) showed the higher amount, followed by gadoleic acid (C20:1n9). Gonçalves et al.^24^ also reported oleic acid as the predominant FA in farmed Chilean salmon samples, with concentrations within the range of 29.8–33.1 g/100 g lipids, being in agreement with the values obtained in the present work. Furthermore, no significant difference (p > 0.05) in MUFA content were found among the samples, regardless of storage condition and storage time.

The highest amount of PUFA was associated to n-6 compounds (10.6–13.3 g/100 g lipids), followed by n-3 compounds in a lower content (6.3–9.3 g/100 g lipids). These results were similar to Gonçalves et al.^24^ for farmed Chilean salmon.

Total n-3 PUFA were mainly composed of eicosatetraenoic acid (C20:4n-3), eicosapentaenoic acid (EPA, C20:5n-3), docosapentaenoic acid (DPA, C22:5n-3) and docosahexaenoic acid (DHA, C22:6n-3), presenting an initial FA content of 0.74 ± 0.10, 1.98 ± 0.16, 0.94 ± 0.11 and 4.51 ± 0.70 g/100 g lipids, respectively (Table 1). From the identified n-3 PUFAs, only DHA showed significant variation (p < 0.05) during storage. HS samples showed tendency to have higher DHA content than the AP samples (mainly at AP/5 °C for 15 days and AP/25 °C for 5 days), with values between 4.24 and 5.39 g/100 g lipids, compared to values between of 3.23 and 4.21 g/100 g lipids for AP samples. DHA is very unstable toward oxidation due to the high number of its double bonds, so for AP samples the decrease of this PUFA might be due to its liability to oxidation at RT (25 °C after 5 days) and under refrigeration (5 °C for longer storage times, 15 days). These results are similar to those reported by Chaijan et al.^25^ and Tenyang et al.^26^ who observed a diminution in PUFA content during refrigerated storage of sardine (*Sardinella gibbosa*) and catfish oil, respectively. The reason for the different results for DHA at AP and HS might be related to the different behaviour of the samples concerning lipid oxidation (see below) and should be further studied. Furthermore, several n-6 PUFAs were detected in the salmon samples: linoleic acid (C18:2n-6), eicosadienoic acid (C20:2n-6) and docosadienoic acid (C22:2n-6), with values for the initial salmon samples of 9.04 ± 1.63, 0.95 ± 0.15 and 2.24 ± 0.45 g/100 g lipids, respectively. Total n-6 PUFAs did not present any significant variation (p > 0.05) for all samples.

Also, no significant changes were observed for the n-6/n-3 ratio (Table 1), with values between 1.38 and 1.88. According to Simopoulos^27^, a lower ratio of n-6/n-3 ratio is beneficial (approximately 1) to decrease the likelihood of many chronic diseases, such as secondary prevention of cardiovascular diseases, reduction of rectal cell proliferation in patients with colorectal cancer, suppression of inflammation in patients with rheumatoid arthritis, and helpful effects on patients with asthma.

**Figure 1:**
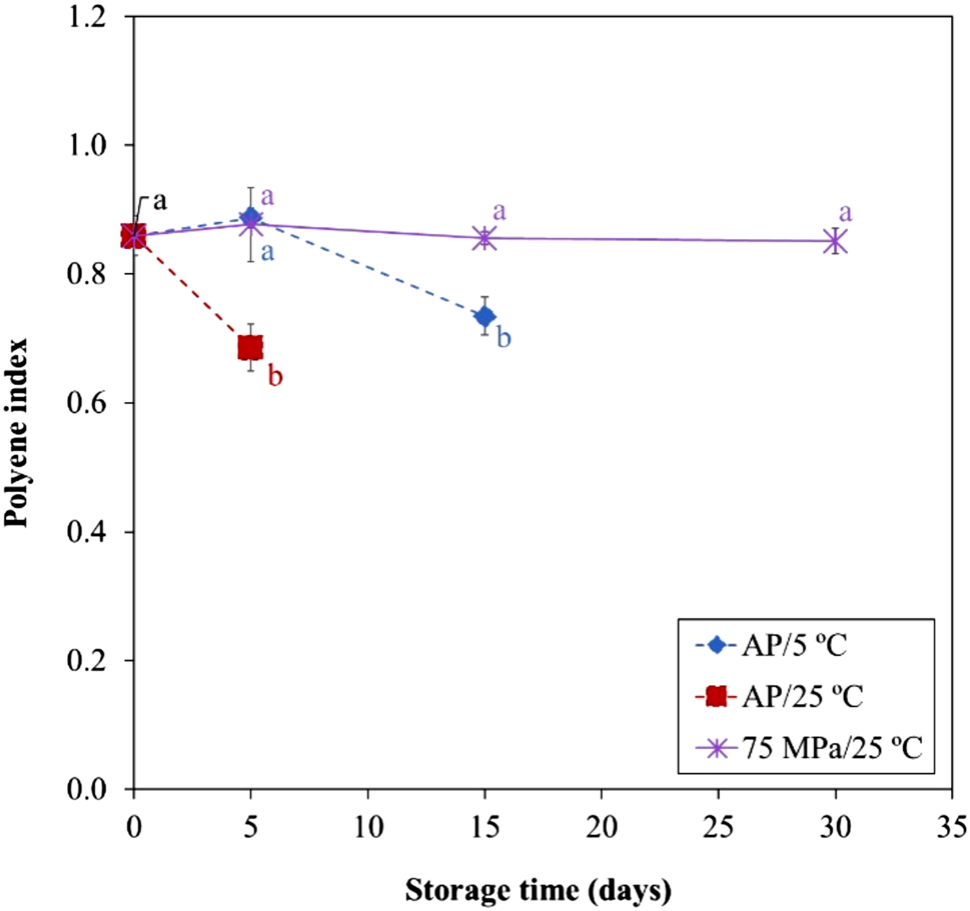
Polyene index of Atlantic salmon stored at 75 MPa/25 °C, AP/5 °C and AP/25 °C during 30, 15 and 5 days, respectively. Different letters denote significant differences (p < 0.05) between salmon samples stored at different conditions and time (**a**,**b**).

Polyene index progress in salmon is a measure of the variation of long chain (LC)‐PUFAs (DHA and EPA) during storage, relative to a saturated fatty acid representative of marine products such as salmon (C16:0), being a good index to evaluate lipid oxidation^13^. Salmon stored at AP/25 °C for 5 days presented already a decrease (p < 0.05) compared to the initial amount of 0.86 ± 0.03 to 0.68 ± 0.04 g/100 g lipids. However, at AP/5 °C, the polyene index decreased also significantly (p < 0.05) but only after 15 days to a value of 0.73 ± 0.03 (Fig. 1). On the other hand, HS did not cause changes (p > 0.05) on polyene index during storage (between 0.85 and 0.88). The reduction of polyene index verified for samples stored under atmospheric pressure indicate that oxidation mechanisms are active in these samples.

No results are available concerning the effect of HS/RT on FA profile of fish products, but recently Otero et al.^9^ reported that hyperbaric cold storage (HS at low temperature, 50 MPa/5 °C during 12 days) showed no considerable alterations on FA composition of Atlantic mackerel during storage, as well as in samples stored at AP/5 °C. These authors verified no changes on polyene index for 12 days, neither at AP/5 °C or at 50 MPa/5 °C^9^. In the present work, most of FA were also not affected by storage conditions; only DHA content was higher after 30 days at HS/RT than at AP, reflecting a higher n-3 PUFAs preservation. Consequently, polyene index was maintained in HS/RT samples, and decreased for AP samples (AP/25 °C after 5 days and AP/5 °C after 15 days).

FA variations under HS/RT were already studied in different food products, such as ready-to-eat cod meal “*Bacalhau com natas”* (50–150 MPa/ ~ 21 °C during 12 h^28^)*,* raw bovine meat (50–150 MPa/ ~ 21 °C during 12 h^29^), and whey cheese (100 MPa/17 °C during 10 days^30^), revealing no consistent pattern to a possible effect of HS/RT, possibly due to the short storage periods (maximum of 10 days).

#### Lipid oxidation evolution

**Figure 2:**
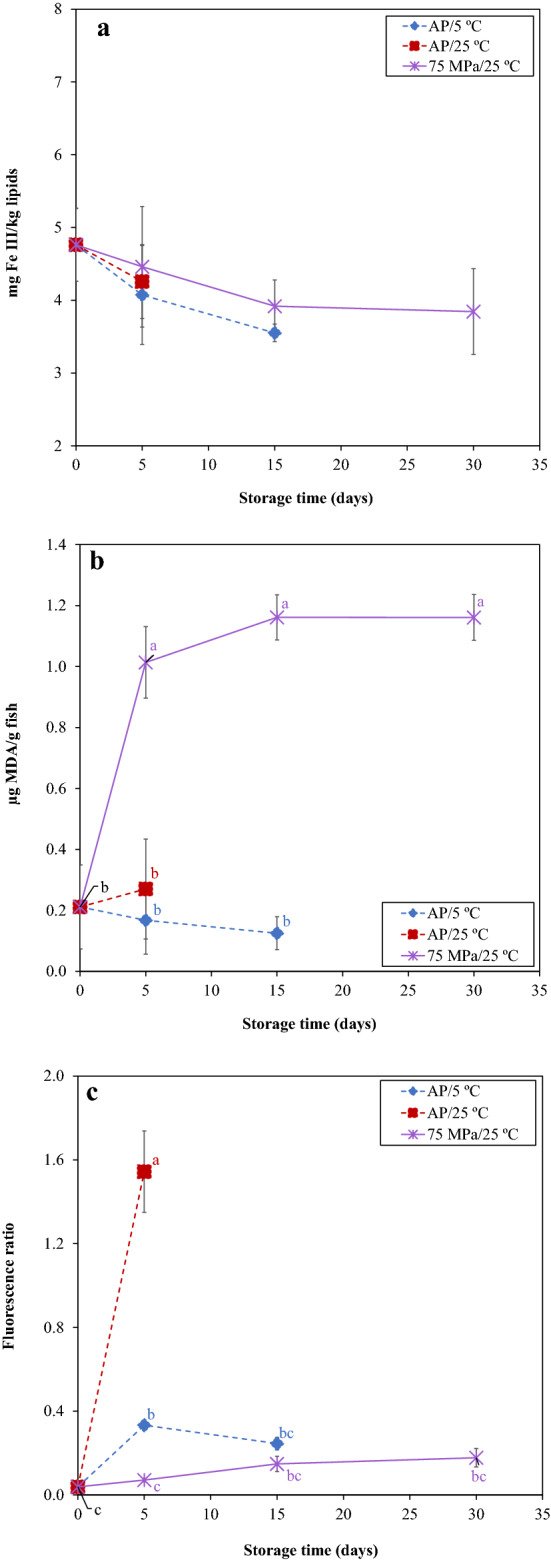
Primary lipid oxidation (**a**; peroxide values in mg Fe III/kg lipids), secondary lipid oxidation (**b**; TBARS in µg MDA/g fish) and tertiary lipid oxidation (**c**; fluorescent compounds in fluorescence ratio) of Atlantic salmon stored at 75 MPa/25 °C, AP/5 °C and AP/25 °C during 30, 15 and 5 days, respectively. Different letters denote significant differences (p < 0.05) between salmon samples stored at different conditions and time (**a**–**c**). For primary lipid oxidation (**a**) no significant differences were observed in all cases.

Primary (peroxide values), secondary (TBARS) and tertiary (fluorescence ratio) lipid oxidation was assessed to evaluate the rancidity development on salmon loins during 30 days of storage (Fig. 2). Initial fresh salmon samples presented peroxide values, TBARS and a fluorescence ratio of 4.76 ± 0.50 mg Fe III/kg lipids, 0.21 ± 0.14 µg MDA/g fish and 0.04 ± 0.01, respectively, in agreement with previous works^7^.

Peroxide values (Fig. 2a) did not show significative changes (p > 0.05) during the 30 days of storage in all conditions, showing values between 3.55 and 4.76 mg Fe III/kg lipids, although with lower numerical values for HS. Concerning to TBARS (Fig. 2b), there was a pronounced increase for samples stored at 75 MPa/25 °C, reaching a value of 1.01 ± 0.12 µg MDA/g fish (fivefold) after 5 days of storage, remaining thereafter constant (p > 0.05) until 30th day. However, a higher increase was observed in previous work^7^, when Atlantic salmon muscle portions were stored at 75 MPa/25 °C, revealing an increment of TBARS values in about 29-fold after 25 days of storage. In the present work, salmon loins were vacuum-packaged, which allowed obtaining lower TBARS values. Lower TBARS values (between 0.13 and 0.27 µg MDA/g fish) were observed for the samples stored under atmospheric pressure (AP/5 and 25 °C, after 15 and 5 days, respectively). Moreover, the fluorescence ratio increased (p < 0.05) for AP samples stored at 25 °C about 40-fold the initial value, showing a fluorescence ratio of 1.54 ± 0.19 after 5 days of storage. For AP/5 °C, there was also a slightly increase (p > 0.05) of about ninefold after 5 days of storage, compared to those obtained in fresh salmon, decreasing after 15 days. For HS, 75 MPa/25 °C, fluorescent compounds did not change (p > 0.05) during 30 days of storage and were much lower compared to the other two storage conditions.

The lipid oxidation mechanism that occur during salmon storage was already described in a previous work^7^. Basically, fish lipid oxidation is a complex chain of reactions, leading to the formation of primary and secondary products that can react with amino constituents (proteins, peptides, free amino acids, and phospholipids), generating interaction compounds (fluorescent compounds). Generally, AP samples (being at 25 °C more pronounced than at 5 °C) showed lower TBARS values and higher fluorescent compounds, as a result of lipid oxidation mechanisms from secondary and tertiary oxidations, respectively. On the other hand, HS samples showed high TBARS values and lower fluorescent compounds, indicating that oxidation extent was lower when compared to AP samples. These results indicate that under HS, no tertiary compounds formation occurs, produced from the interaction between secondary products and amino constituents of the muscle, resulting in higher TBARS but a lower fluorescence ratio value.

### Protein stability–myofibrillar fragmentation index

**Figure 3:**
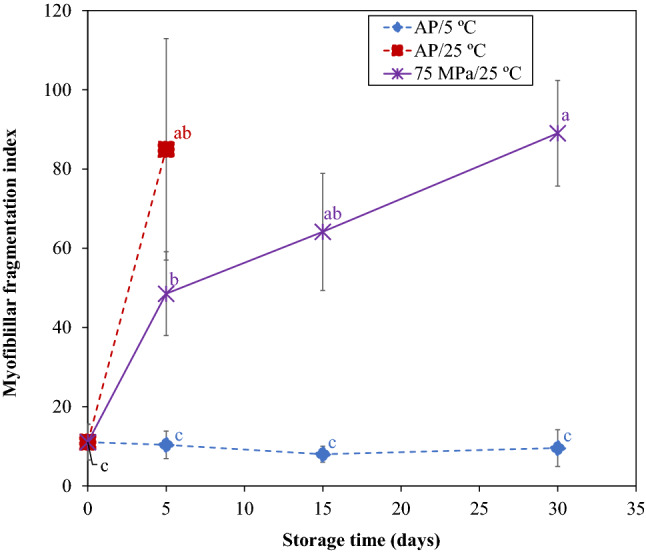
Myofibrillar fragmentation index of Atlantic salmon stored at 75 MPa/25 °C and AP/5 °C during 30 days, and at AP/25 °C during 5 days. Different letters denote significant differences (p < 0.05) between salmon samples stored at different conditions and time (**a**–**c**).

Myofibrillar proteins are the main constituents of muscle being intensily degraded by proteolysis a few days after *postmortem*. Myofibrillar fragmentation index (MFI) is indicative of the degree of muscle myofibrillar protein degradation, being an useful indicator of I-band (composed by actin) separation and breakage of intermyofibrils linkages^31^. As shown in Fig. 3, MFI salmon samples significantly increased (p > 0.05) after 5 days of storage at AP/25 °C, from 11 ± 4 to 85 ± 28, which correspond to an increase of about 7.7-fold the initial value. At 75 MPa/25 °C, MFI also increased (p < 0.05) after 30 days of storage (89 ± 13), reaching values similar (p > 0.05) as at AP/25 °C after 5 days. However, no changes (p > 0.05) were verified at AP/5 °C after 30 days of storage, showing a MFI value of 10 ± 5 at the end of storage. Although for a slight higher temperature, different results were obtained by Wang et al.^32^ for grass carp (*Ctenopharyngodon idellus*), with MFI significantly increasing during storage for 8 h at 8 °C (under AP conditions).

Otero et al.^9^ reported that HS at low temperature (50 MPa/5 °C) led to differences on the electrophoretic pattern of the myofibrillar fraction of Atlantic mackerel, what could be due to a direct effect of pressure denaturation on myofibrillar proteins, but also to a pressure-induced effect on the autolytic capacity of endogenous proteases. In the present work, there was no effect in MFI for AP/5 °C samples after 30 days of storage, but a pronounced effect of storage at AP/25 °C was verified, showing an increase of about eightfold the initial MFI. At 75 MPa/25 °C, after 5 days salmon samples revealed a slower increase of about fourfold. However, after 15 days, an increase of about sixfold, resulting in similar values to AP/25 °C (5 days), after 30 days of storage (eightfold). This behaviour can be explained by a possibly inhibition of autolytic enzymes (proteases) under pressure, reflecting in a lower deteriorative effect on muscle structure.

### Volatile compounds identification

Twenty-nine volatile compounds were detected in the headspace over salmon samples using SPME-GC/MS analysis (Table 2). The compounds identified were mainly alcohols (n = 6), aldehydes (n = 10), alkanes (n = 7), esters (n = 4), ketones (n = 1) and sulphur compounds (n = 1).

**Table 2:** Volatile compounds Atlantic salmon stored at 75 MPa/25 °C and AP/5 °C during 30 days, and at AP/25 °C during 5 days.

Volatile compounds	RT (min)	KI	Fresh fish	75 MPa/25 °C			AP/5 °C			AP/25 °C
			0 days	6 days	15 days	30 days	5 days	15 days	30 days	5 days
**Alcohols**										
Pent-1-en-3-ol	2.921	670	0.073** ± **0.006	0.081** ± **0.009	0.086** ± **0.047	0.031** ± **0.006	0.074** ± **0.027	0.060** ± **0.016	0.068** ± **0.005	nd
3-Methylbutan-1-ol	3.847	717	nd	nd	nd	nd	0.038** ± **0.011^d^	0.969** ± **0.066^a^	0.497** ± **0.022^b^	0.157** ± **0.074^c^
2-Methylbutan-1-ol	3.954	720	nd	nd	nd	nd	0.009** ± **0.006^c^	0.202** ± **0.030^a^	0.255** ± **0.058^a^	0.084** ± **0.027^b^
Butane-2,3-diol	5.913	779	nd	nd	nd	nd	0.065** ± **0.032^b^	0.145** ± **0.009^a^	0.090** ± **0.037^ab^	0.108** ± **0.044^ab^
Oct-1-en-3-ol	12.930	973	0.064** ± **0.063	0.078** ± **0.016	0.076** ± **0.038	0.039** ± **0.003	0.052** ± **0.019	0.057** ± **0.021	0.055** ± **0.004	nd
Phenylethyl alcohol	19.375	1108	nd	nd	nd	nd	0.014** ± **0.003^c^	0.228** ± **0.008^a^	0.136** ± **0.035^b^	0.222** ± **0.028^a^
**Aldehydes**										
3-Methylbutanal	2.569	642	nd	nd	nd	0.022** ± **0.007^b^	0.021** ± **0.007^b^	0.095** ± **0.042^a^	0.072** ± **0.018^a^	nd
Hexanal	5.533	768	0.362** ± **0.123^a^	0.234** ± **0.043^abc^	0.164** ± **0.081^bc^	0.100** ± **0.034^c^	0.340** ± **0.075^ab^	0.248** ± **0.024^abc^	0.404** ± **0.062^a^	0.113** ± **0.002^c^
Hept-4-enal	9.305	893	0.059** ± **0.010^a^	0.046** ± **0.011^ab^	0.026** ± **0.007^bc^	0.012** ± **0.005^c^	0.044** ± **0.003^ab^	0.038** ± **0.004^b^	0.042** ± **0.010^ab^	nd
Heptanal	9.428	898	0.067** ± **0.035^a^	0.025** ± **0.008^ab^	0.034** ± **0.025^ab^	0.011** ± **0.006^b^	0.037** ± **0.008^ab^	0.051** ± **0.012^ab^	0.049** ± **0.013^ab^	nd
Benzaldehyde	11.778	951	0.060** ± **0.036^b^	0.052** ± **0.019^b^	0.060** ± **0.026^ab^	0.045** ± **0.002^b^	0.042** ± **0.026^b^	0.104** ± **0.034^ab^	0.133** ± **0.018^a^	0.076** ± **0.029^ab^
Octanal	14.104	1004	0.023** ± **0.006^b^	0.070** ± **0.019^a^	0.059** ± **0.025^ab^	0.023** ± **0.008^b^	0.046** ± **0.016^ab^	0.046** ± **0.017^ab^	0.078** ± **0.011^a^	nd
Hepta-2,4-dienal	14.420	1005	0.087** ± **0.021	0.062** ± **0.034	0.100** ± **0.072	nd	0.105** ± **0.012	0.098** ± **0.027	0.060** ± **0.022	nd
Phenylacetaldehyde	15.747	1032	nd	nd	nd	nd	0.024** ± **0.014^c^	1.363** ± **0.306^a^	0.483** ± **0.073^b^	0.310** ± **0.161^bc^
Nonanal	19.044	1101	0.029** ± **0.009	0.062** ± **0.014	0.087** ± **0.057	0.056** ± **0.014	0.064** ± **0.019	0.078** ± **0.025	0.065** ± **0.016	0.087** ± **0.012
Decanal	23.852	1200	0.019** ± **0.020	0.022** ± **0.008	0.022** ± **0.018	0.012** ± **0.007	0.019** ± **0.008	0.022** ± **0.007	0.011** ± **0.002	0.024** ± **0.012
**Alkanes**										
Decane	13.961	1000	0.096** ± **0.026^b^	0.276** ± **0.090^a^	0.103** ± **0.047^b^	0.087** ± **0.038^b^	0.205** ± **0.030^ab^	0.157** ± **0.054^ab^	0.104** ± **0.017^b^	nd
Undecane	18.644	1093	0.120** ± **0.025	0.450** ± **0.156	0.246** ± **0.159	0.207** ± **0.060	0.291** ± **0.062	0.204** ± **0.059	0.136** ± **0.014	0.356** ± **0.245
Dodecane	23.313	1190	0.076** ± **0.013	0.293** ± **0.116	0.196** ± **0.116	0.199** ± **0.043	0.159** ± **0.038	0.111** ± **0.020	0.075** ± **0.009	0.239** ± **0.156
Tridecane	27.240	1290	0.039** ± **0.008	0.133** ± **0.057	0.088** ± **0.053	0.099** ± **0.020	0.066** ± **0.017	0.041** ± **0.001	0.028** ± **0.001	0.113** ± **0.076
Pentadecane	33.403	1496	0.097** ± **0.034^c^	0.060** ± **0.025^c^	0.069** ± **0.032^c^	0.055** ± **0.025^c^	0.101** ± **0.047^c^	0.134** ± **0.055^bc^	0.421** ± **0.079^a^	0.249** ± **0.114^b^
Heptadecane	37.830	1680	0.107** ± **0.065^bcd^	0.059** ± **0.033^d^	0.075** ± **0.056^ cd^	0.051** ± **0.028^d^	0.083** ± **0.044^bcd^	0.080** ± **0.012^bc^	0.088** ± **0.015^b^	0.148** ± **0.025^a^
2,6,10,14-Tetramethylpentadecane	38.335	1700	0.164** ± **0.037^b^	0.093** ± **0.003^b^	0.111** ± **0.050^b^	0.085** ± **0.006^b^	0.135** ± **0.043^b^	0.180** ± **0.032^b^	0.080** ± **0.009^b^	0.352** ± **0.062^a^
**Esters**										
Ethyl acetate	2.256	616	0.025** ± **0.001^bc^	nd	nd	nd	0.127** ± **0.016^bc^	0.324** ± **0.113^ab^	0.325 ± 0.113^ab^	0.554** ± **0.279^a^
Ethyl 2-methylpropanoate	4.458	735	nd	nd	nd	nd	nd	nd	nd	0.560** ± **0.126
Ethyl 2-methylbutanoate	7.356	826	nd	nd	nd	nd	nd	nd	nd	0.866** ± **0.302
Ethyl 4-methylpentanoate	12.412	962	nd	nd	nd	nd	nd	nd	nd	2.815** ± **0.591
**Ketones**										
Butan-2-one	2.141	607	nd	nd	nd	nd	0.021** ± **0.006^b^	0.019** ± **0.009^b^	0.021** ± **0.008^b^	1.312** ± **0.663^a^
**Sulphur compounds**										
Dimethyl disulphide	4.098	724	nd	nd	nd	nd	nd	nd	nd	0.164** ± **0.023

Different letters along each row denote significant differences (p < 0.05); absence of letters indicates not statistically significant differences. Values correspond to the peak area of each compound/peak area of internal standard ratio.

*KI* experiment value of Kovats Index (KI).

A total of six alcohols were identified, being only pent-1-en-3-ol and oct-1-en-3-ol present on fresh salmon (Table 2). These two compounds were not detected in samples stored at AP/25 °C. Generally, these alcohols are used as markers for salmon freshness, and are originated from n-6 PUFA oxidation by lipoxygenase and/or other chemical reactions^33^. Their presence in fish might originate from the degradation of linoleic acid and α-linolenic acid, respectively^34^. Other alcohols, such as phenylethyl alcohol, 2-methylbutan-1-ol, 3-methylbutan-1-ol and butane-2,3-diol, were only detected in samples stored at AP (5 and 25 °C). These compounds are associated to microbial activity and are considered as possible indicators of fish spoilage or loss of freshness during storage^35^.

In total, ten aldehydes were identified by SPME-GC/MS during storage (Table 2). Among them, hexanal, nonanal, decanal and benzaldehyde were found in all samples stored at the different conditions. Other aldehydes detected in salmon samples, such as heptanal, octanal, hept-4-enal and hepta-2,4-dienal were not detected at AP/25 °C. According to the literature, these C6–C9 aldehydes are commonly described as the main compounds responsible of typical fresh-fish flavour^36^, being produced from lipoxygenase action on n-6 or n-9 PUFAs^37^. For instance, formation of hexanal and heptanal has been reported as being originated from the oxidation of n-6 PUFA (mainly from linoleic acid) and that of nonanal from n-9 PUFA oxidation (mainly from oleic acid)^37^. In contrast, phenylacetaldehyde was only detected in samples stored at AP/5 and 25 °C, and 3-methylbutanal in samples stored at AP/5 °C and 75 MPa/25 °C (only after 30 days). The production of 3-methylbutanal and phenylacetaldehyde has been shown to be related to spoilage and could originate from both Strecker degradation and microbial activity on leucine and phenylalanine, respectively, formed by the reaction between α-dicarbonyl compounds and amino acids^38,39^.

Alkanes were also significantly present in the volatiles of salmon samples. Seven alkanes were identified in all samples, such as decane, undecane, dodecane, tridecane, pentadecane, heptadecane and pristane (2,6,10,14-tetramethylpentadecane); only decane was not detected in samples stored at AP/25 °C after 5 days. It has been reported that C8–C19 alkanes contribute only slightly to the flavour because they present high odour threshold values^40^. Pristane (2,6,10,14-tetramethylpentadecane) is a naturally occurring hydrocarbon produced by copepods, while other organisms fed on copepods and do not readily metabolize this compound, causing it to accumulate through the marine food chain^41^.

Four ester compounds were identified in salmon samples: ethyl acetate, ethyl 2-methylpropanoate, ethyl 2-methylbutanoate and ethyl 4-methylpentanoate. Ethyl acetate was identified in fresh fish in small quantities and in samples stored at AP, being present in high quantities in samples stored at AP/25 °C. Moreover, ethyl 2-methylpropanoate, ethyl 2-methylbutanoate and ethyl 4-methylpentanoate were detected only in samples stored at AP/25 °C. Ethyl esters formation was associated with esterase activity of lactic acid bacteria^42^ and amino acid catabolism of *Pseudomonas* ssp.^43^.

Only one ketone was detected in salmon samples, butan-2-one, being present in samples stored at AP, and at an higher amount in samples stored at 25 °C. In addition, dimethyl disulphide was only detected at AP/25 °C. Dimethyl disulphide can be formed by endogenous enzymes action and by microbial activity, and has been suggested as a main cause of putrid spoilage aromas in fish^44^. These compounds have been associated to the action of H_2_S-producing organisms, like *Shewanella putrefaciens*, thus contributing to off-flavour in fish products^45^. These sulphur compounds originated from microbial degradation of cysteine and methionine to form hydrogen sulphide and methyl mercaptan, respectively^46^. The mechanism proposed to produce oxidized volatile sulphur compounds includes the fast air oxidation of methyl mercaptan to form dimethyl disulphide^47^.

Overall, several potential markers for salmon freshness (pent-1-en-3-ol, oct-1-en-3-ol, hexanal, heptanal, octanal, nonanal, decanal, benzaldehyde, hept-4-enal, hepta-2,4-dienal, alkanes) and for spoilage of salmon (phenylethyl alcohol, 2-methylbutan-1-ol, 3-methylbutan-1-ol, butane-2,3-diol, phenylacetaldehyde, 3-methylbutanal, ethyl esters, butan-2-one, dimethyl disulphide) were identified, showing clear differences between HS and AP preserved samples. The volatile profile of samples stored at 75 MPa/25 °C was more similar to fresh ones, retaining fresh-like alcohols and aldehyde components, for example pent-1-en-3-ol, oct-1-en-3-ol, heptanal, octanal and hept-4-enal, which were not detected or detected at lower amounts for samples stored at AP/25 °C and AP/5 °C particularly for the former. The majority of these fresh-like compounds were not detected in samples stored at AP (5 °C and 25 °C), but spoilage-like compounds of microbial activity (phenylethyl alcohol, 2-methylbutan-1-ol, 3-methylbutan-1-ol, butane-2,3-diol, ethyl esters and dimethyl disulphide) were found instead.

### Physical properties

#### Drip loss and water holding capacity

After 5 days of storage, salmon samples stored at AP/25 °C showed a drip loss of 9.95%, while for HS (75 MPa/25 °C) and AP/5 °C, drip loss presented lower values of 6.09% and 1.44%, respectively (Table 3). During storage, drip loss increased (p < 0.05) in samples at these two conditions (75 MPa/25 °C and AP/5 °C). At 75 MPa/25 °C, drip loss increased progressively, with a linear change tendency (Drip loss = 0.2946 × storage time (days) + 4.451, r^2^ = 0.9957) reaching a higher value (p < 0.05) of 13.40% after 30 days of storage, compared to the value of 6.62% at AP/5 °C.

**Table 3:** Physical properties of Atlantic salmon stored at 75 MPa/25 °C and AP/5 °C during 30 days, and at AP/25 °C during 5 days.

Physical property	Fresh fish	75 MPa/25 °C			AP/5 °C			AP/25 °C
	0 days	5 days	15 days	30 days	5 days	15 days	30 days	5 days
Water content (%)	71.8 ± 3.2	70.5 ± 0.9	71.5 ± 0.3	69.5 ± 3.5	69.8 ± 1.8	69.4 ± 3.0	73.3 ± 4.3	67.0 ± 1.2
Drip loss (%)	–	6.1 ± 1.0^bcd^	8.6 ± 3.3^ab^	13.4 ± 2.4^a^	1.4 ± 0.3^d^	3.8 ± 1.3^ cd^	6.6 ± 1.0^bc^	10.0 ± 0.7^ab^
Water holding capacity (%)	95.8 ± 0.7^ab^	92.2 ± 1.8^c^	94.7 ± 1.0^abc^	96.4 ± 0.7^ab^	96.5 ± 0.5^ab^	93.8 ± 0.9^bc^	97.9 ± 0.7^a^	93.6 ± 2.2^bc^
**Texture**								
Hardness (N)	3.21 ± 0.51	2.22 ± 0.22	2.15 ± 0.72	2.29 ± 0.38	2.74 ± 0.39	3.27 ± 0.28	2.43 ± 0.41	2.67 ± 0.43
Adhesiveness (N s)	-0.36 ± 0.11	-0.25 ± 0.09	-0.48 ± 0.16	-0.33 ± 0.05	-0.43 ± 0.12	-0.44 ± 0.13	-0.38 ± 0.18	-0.21 ± 0.00
Springiness	1.49 ± 0.12	1.28 ± 0.28	1.22 ± 0.11	1.14 ± 0.24	1.37 ± 0.28	1.38 ± 0.10	1.42 ± 0.14	1.13 ± 0.07
Resilience	1.27 ± 0.29^a^	0.70 ± 0.57^ab^	0.63 ± 0.10^ab^	0.09 ± 0.01^b^	0.99 ± 0.65^ab^	0.95 ± 0.19^ab^	0.95 ± 0.21^ab^	0.45 ± 0.20^ab^

Different letters along each row denote significant differences (p < 0.05); absence of letters indicates not statistically significant differences.

The initial water holding capacity (WHC) was 95.8%, which is in agreement with values obtained by other authors for Atlantic salmon^48,49^. At AP/5 °C, WHC did not change (p > 0.05) during the 30 days, neither under storage at AP/25 °C during 5 days; while at 75 MPa/25 °C, a slight decrease (p < 0.05) after 5 days was observed to a value of 92.2%, increasing further to a value of 96.4% after 30 days, with a linear change tendency (WHC = 0.4215 × storage time (days) + 90.213, r^2^ = 0.9857).

Drip loss is a factor that adversely influence the consumers acceptability of muscle foods. Generally, high pressure at levels used for pasteurization (400–600 MPa) during few minutes, promotes the drip loss and reduces WHC, which is primarily related to disturbance of proteins electrostatic and hydrophobic interactions, inducing alterations in protein–protein conformation and denaturation of important myofibrillar and/or sarcoplasmic proteins^50^, resulting in exudation from muscles, resulting in drip loss. This behaviour was observed in samples stored at 75 MPa/25 °C, since drip loss increased during the 30 days of storage. Furthermore, Christensen et al.^48^ stated that denaturation of structural proteins, like myosin and actin, induced by pressure, caused reduced WHC of the proteins, in accordance to what was observed in the current study after 5 days at 75 MPa/25 °C. However, WHC increased during storage under pressure, possibly due to the effect of compression leading to the expulsion of most of free fluid from the fish muscle, which reduced the amount of expressed soluble proteins and increased the WHC of muscle^51^. In addition, there was a linear correlation between drip loss and WHC (WHC = 0.5459 × Drip loss + 89.319; r^2^ = 0.9123) for HS samples. Otero et al.^8^ stored Cape hake loins at 50 MPa/5 °C and verified a comparable behaviour; WHC of HS samples increased after 7 days, with the authors explaining these results, due to that fact that possibly part of the free water in these samples had been released before due to drip loss and the reminiscent water was more strongly retained by the tissue.

#### Texture properties

Textural properties were evaluated by texture profile analysis (TPA) that mimics mastication or chewing process, being performed with double-compression cycles, allowing the quantification of a wide range of texture properties: hardness, adhesiveness, springiness and resilience (Table 3).

Surprisingly, considering the effects discussed above, no significant differences (p > 0.05) were found for the textural properties of hardness, adhesiveness and springiness for all the samples studied. However, resilience decreased (p < 0.05) from 1.27 ± 0.29 to 0.09 ± 0.01 for samples stored at 75 MPa/25 °C after 30 days. Resilience measures muscle elasticity being related with the ability of the muscle to recover from deformation and the resistance to a subsequent deformation^52^. Veland and Torrissen^52^ stated that if resilience is equal to 1, the work performed by the probe during the downstroke is returned by the muscle during the upstroke. In the case of HS samples seemed that fibres affected by the deformation would not all be stretched at the same rate and would not all reach the limit of elastic (recoverable) deformation at the same time. Fish muscle proteins are essential for quality characteristics such as textural properties; although no significant effects were observed for other textural characteristics such as hardness, adhesiveness and springiness as discussed, increase of MFI for these samples may be correlated to the low resilience values.

Chéret et al.^53^ verified that TPA parameters are diversely affected by higher pressure, and its effect changes markedly at about 300 MPa, for 5 min. However, a lower pressure (75 MPa) was used in the present work and for longer times (up to 30 days). Cape hake loins^8^ and Atlantic mackerel^9^ were stored at 50 MPa/5 °C during 7 and 12 days, respectively, being verified that the shear resistance of Cape hake loins increased, but in the case of Atlantic mackerel, no effect on firmness was observed.

## Conclusions

HS/RT (75 MP/25 °C) of vacuum-packaged fresh Atlantic salmon was found to be an efficient method to retain important physicochemical properties up to at least 15 days of storage. Most of the FAs were not changed by storage conditions and the main difference was observed for DHA that showed no changes for samples stored under HS/RT during the 30 days, but decreased for samples stored under AP, reflecting in a stable and better n-3 PUFAs content. Accordingly, polyene index was maintained in HS/RT samples, and decreased for AP samples (AP/25 °C after 5 days and AP/5 °C after 15 days). Furthermore, HS samples revealed a slower increase of MFI values and lower lipid oxidation when compared to AP samples. The volatile compounds identified were mainly alcohols, aldehydes, alkanes, esters, ketones and sulphur compounds. Samples stored at 75 MPa/25 °C showed a similar volatile profile to that of fresh samples. On the other hand, spoilage-like compounds were detected in higher amounts in samples stored under AP. Drip loss increased during storage under pressure, with no significant differences on WHC after 30 days of storage. No effect of HS was found for the textural properties of hardness, adhesiveness and springiness, while resilience decreased for samples stored at 75 MPa/25 °C after 30 days.

Overall, the results point for HS/RT as an interesting methodology for fresh fish extended preservation, resulting in equal to better physicochemical parameters, with the additional feature of allowing significant energy savings, since energy, because it is only used for compression and decompression, since pressure maintenance during storage needs no energy.

The commercial implementation of HS needs to be analyzed further as for any novel technology, in questions related to equipments development, as well as more studies, like sensorial and microbiological studies with pathogenic microorganisms.

## Supplementary Information

Supplementary Information.

## Data Availability

The data that support the findings of this study are available on request from the corresponding author (J.A. Saraiva).
