# Supplementary material for: Enhanced preservation of vacuum-packaged Atlantic salmon by hyperbaric storage at room temperature versus refrigeration
Source: Sci Rep. 2021 Jan 18;11:1668. doi: 10.1038/s41598-021-81047-4 (PMC7814072; doi:10.1038/s41598-021-81047-4)
Supplement: Supplementary file 1 — Supplementary Information. [file 41598_2021_81047_MOESM1_ESM.pdf]

Enhanced preservation of vacuum-packaged Atlantic salmon by hyperbaric storage at room temperature versus refrigeration

Liliana G. Fidalgo[1], Mário M. Q. Simões[1], Susana Casal[2], José A. Lopes-da-Silva[1], Ivonne Delgadillo[1], Jorge A. Saraiva[1*]

Supplementary Table

Table 1S – Fatty acids (FA) profile (wt.%, g FA/100 g of total lipids) along storage of Atlantic salmon stored at 75 MPa/25 ºC, AP/5 ºC and AP/25 ºC for different storage times. Different letters along each row denote significant differences ($p < 0.05$) and absence of letters indicates not statistically significant differences.

Free fatty acids [(1)]	*75 MPa/25 ºC*			*AP/5 ºC*		*AP/25 ºC*
	5 days	***15 days***	***30 days***	***5 days***	***15 days***	***5 days***
C14:0	1.27 ± 0.13	1.17 ± 0.06	1.05 ± 0.19	1.01 ± 0.10	1.30 ± 0.14	1.14 ± 0.02
C16:0	8.71 ± 0.61	8.24 ± 0.50	7.15 ± 1.41	6.78 ± 0.97	7.37 ± 0.16	7.86 ± 1.29
C18:0	1.57 ± 1.14	2.06 ± 0.12	1.82 ± 0.30	1.84 ± 0.18	1.91 ± 0.20	2.02 ± 0.35
∑ SFA	**11.55 ± 1.02**	**11.47 ± 0.58**	**10.02 ± 1.90**	**9.62 ± 1.17**	**10.57 ± 0.36**	**10.01 ± 2.08**
C16:1n-7	1.59 ± 0.24	1.57 ± 0.13	1.22 ± 0.16	1.34 ± 0.09	1.68 ± 0.12	1.49 ± 0.09
C18:1n-7	3.70 ± 0.31	3.09 ± 0.11	2.99 ± 1.09	3.34 ± 0.76	3.54 ± 0.81	3.76 ± 0.14
C18:1n-9	28.06 ± 3.29	29.80 ± 1.62	28.59 ± 1.06	29.69 ± 3.20	31.47 ± 5.33	28.53 ± 1.97
C20:1n-9	4.79 ± 0.23	4.46 ± 0.15	3.90 ± 0.70	4.36 ± 0.55	4.89 ± 0.74	4.62 ± 0.67
C22:1n-9	0.80 ± 0.13	0.71 ± 0.02	0.71 ± 0.18	0.66 ± 0.11	0.80 ± 0.21	0.76 ± 0.04
∑ MUFA	**38.94 ± 4.13**	**39.63 ± 1.83**	**37.42 ± 1.09**	**39.39 ± 4.40**	**42.38 ± 6.64**	**37.72 ± 2.76**
C20:4n-3	0.76 ± 0.04	0.74 ± 0.07	0.64 ± 0.05	0.66 ± 0.06	0.71 ± 0.08	0.72 ± 0.13
C20:5n-3 (EPA)	2.24 ± 0.29	2.12 ± 0.12	1.86 ± 0.36	1.80 ± 0.27	1.90 ± 0.05	2.01 ± 0.46
C22:5n-3	0.94 ± 0.12	0.93 ± 0.04	0.86 ± 0.16	0.90 ± 0.08	0.95 ± 0.07	0.87 ± 0.11
C22:6n-3 (DHA)	5.39 ± 0.41^{a}	4.93 ± 0.34^{ab}	4.24 ± 0.92^{abc}	4.21 ± 0.65^{abc}	3.52 ± 0.32^{bc}	3.23 ± 0.55^{c}
∑ n-3 PUFA	$\mathbf{9.33 \pm 0.76^{a}}$	$\mathbf{8.72 \pm 0.40^{ab}}$	$\mathbf{7.59 \pm 1.48^{ab}}$	$\mathbf{7.57 \pm 0.99^{ab}}$	$\mathbf{7.08 \pm 0.40^{ab}}$	$\mathbf{6.31 \pm 1.26^{b}}$
C18:2n-6	9.49 ± 0.50	8.87 ± 0.27	7.78 ± 1.25	8.35 ± 1.10	9.86 ± 1.49	9.11 ± 0.69
C20:2n-6	0.97 ± 0.03	0.92 ± 0.04	0.83 ± 0.15	0.89 ± 0.06	0.99 ± 0.11	0.93 ± 0.05
C22:2n-6	2.31 ± 0.08	2.24 ± 0.18	2.02 ± 0.21	2.14 ± 0.10	2.43 ± 0.42	2.16 ± 0.18
∑ n-6 PUFA	**12.77 ± 0.59**	**12.02 ± 0.48**	**10.63 ± 1.60**	**11.37 ± 1.25**	**13.28 ± 2.00**	**11.26 ± 1.76**
PUFA/SFA	**1.93 ± 0.22**	**1.81 ± 0.10**	**1.82 ± 0.04**	**1.97 ± 0.06**	**1.92 ± 0.14**	**1.77 ± 0.07**
n-6/n-3 ratio	**1.38 ± 0.16**	**1.38 ± 0.10**	**1.41 ± 0.08**	**1.51 ± 0.09**	**1.88 ± 0.30**	**1.79 ± 0.14**

(1) SFA: total saturated fatty acid; MUFA: monounsaturated fatty acid; PUFA: polyunsaturated fatty acid; EPA: Eicosapentaenoic acid; DHA: Docosahexaenoic acid.
